# An Update on Maternal Hydration Strategies for Amniotic Fluid Improvement in Isolated Oligohydramnios and Normohydramnios: Evidence from a Systematic Review of Literature and Meta-Analysis

**DOI:** 10.1371/journal.pone.0144334

**Published:** 2015-12-11

**Authors:** Salvatore Gizzo, Marco Noventa, Amerigo Vitagliano, Andrea Dall’Asta, Donato D’Antona, Clive J. Aldrich, Michela Quaranta, Tiziana Frusca, Tito Silvio Patrelli

**Affiliations:** 1 Department of Woman and Child Health – University of Padua, Padua, Italy; 2 Department of Obstetrics and Gynecology - NHS Trust - Northampton General Hospital– Northampton, United Kingdom; 3 Department of Surgical Sciences – University of Parma, Parma, Italy; 4 Department of Obstetrics and Gynaecology, University of Verona, Verona, Italy; 5 Vicenza General Hospital, Vicenza, Italy; Shanghai 1st Maternity and Infant hospital of Tongji University, CHINA

## Abstract

**Objective:**

Several trials aimed at evaluating the efficacy of maternal hydration (MH) in increasing amniotic-fluid-volume (AFV) in pregnancies with isolated oligohydramnios or normohydramnos have been conducted. Unfortunately, no evidences support this intervention in routine-clinical-practice. The aim of this systematic-literature-review and meta-analysis was to collect all data regarding proposed strategies and their efficacy in relation to each clinical condition for which MH-therapy was performed with the aim of increasing amniotic-fluid (AF) and improving perinatal outcomes.

**Materials and Methods:**

A systematic literature search was conducted in electronic-database MEDLINE, EMBASE, ScienceDirect and the Cochrane-Library in the time interval between 1991 and 2014. Following the identification of eligible trials, we estimated the methodological quality of each study (using QADAS-2) and clustered patients according to the following outcome measures: route of administration (oral versus intravenous versus combined), total daily dose of fluids administered (<2000 versus >2000), duration of hydration therapy: (1 day, >1 day but <1 week, >1 week), type of fluid administered (isotonic versus hypotonic versus combination).

**Results:**

In isolated-oligohydramnios (IO), maternal oral hydration is more effective than intravenous hydration and hypotonic solutions superior to isotonic solutions. The improvement in AFV appears to be time-dependent rather than daily-dose dependent. Regarding normohydramnios pregnancies, all strategies seem equivalent though the administration of hypotonic-fluid appears to have a slightly greater effect than isotonic-fluid. Regarding perinatal outcomes, data is fragmentary and heterogeneous and does not allow us to define the real clinical utility of MH.

**Conclusions:**

Available data suggests that MH may be a safe, well-tolerated and useful strategy to improve AFV especially in cases of IO. In view of the numerous obstetric situations in which a reduced AFV may pose a threat, particularly to the fetus, the possibility of increasing AFV with a simple and inexpensive practice like MH-therapy may have potential clinical applications. Considering the various strategies of maternal hydration implemented in the treatment of IO, better results were observed when treatment was based on a combination of intravenous (for a period of 1 day) and oral (for a period of at least 14 days) hypotonic fluids (≥2000ml).

## Introduction

Amniotic fluid (AF) is maintained in a dynamic equilibrium and its volume derived from the sum of inflow (from fetal urine and lung fluid) and outflow (fetal swallowing and intramembranous absorption) of fluid from the amniotic space.[1] Amniotic fluid volume (AFV) is an important parameter in the assessment of fetal wellbeing since it provides a number of functions vital to fetal development such as a supportive environment for growth,, protection from trauma and infection and a medium which allows fetal movement thus promoting the development of the musculoskeletal system. AF also prevents a possible compression of the umbilical cord and placenta thereby protecting the fetus from vascular and nutritional compromise.[2]

To date several methods are used to assess AFV ranging from subjective assessment (where the volume is described as average, above average, below average or scant) to semi-quantitative estimations including measurement of the deepest vertical pocket and amniotic fluid index (AFI).[3]

While debate continues regarding the best method to estimate AFV, it has become evident that there are numerous maternal and fetal risk factors associated with a reduction of this parameter. [4–8] Indeed, an increased fetal and neonatal morbidity and mortality is described when pregnancies are complicated by oligohydramnios.[9,10]

Generally about 3–5% of pregnancies are complicated by oligohydramnios, and in less than half of the cases, the diagnosis is made in the absence of maternal-fetal risk factors and is therefore defined “isolated oligohydramnios” (IO).[11]Nevertheless, the lack of a comprehensive understanding of the physiology and dynamics of IO contribute to the unresolved dilemma.[11]

Emerging evidences suggest that oligohydramnios is only a weak predictor of poor perinatal outcome,[12] and, while in term pregnancies an appropriate threshold for intervention seems to be good medical practice,[13] in preterm IO conservative management and strict follow up are justified and should be considered gold standard.

Occasionally IO may be diagnosed in pregnant women with a personal history of insufficient fluid intake. This fact triggered speculation regarding the potential role of maternal dehydratation in contributing to the development of this “borderline condition”.[14] Maternal hydration (MH) has been proposed as a possible effective treatment for the conservative management of IO during pregnancy and prior to labour commencing.[15]

Several trials have been conducted [14–29] to evaluate the efficacy of MH but the heterogeneity in patient selection criteria, sonographic diagnostic criteria, implementation of different hydration protocols and outcomes measured generated considerable confusion in defining the utility of maternal hydration.[30,31] Despite the feeling that simple MH may increase AFV and be beneficial in the management of oligohydramnios, the last of two meta-analysis conducted concluded that further controlled trials are needed to assess the clinical benefits and possible risks of MH for specific clinical purposes.[32]

Authors evaluated the effects of MH in patients with normal AFV which may be of interest in obstetric care since the bid to reduce the rate of unnecessary caesarean section compels us to search for new strategies to facilitate vaginal deliveries.[33–36] The amount of AF is associated with the success of external cephalic version [37] and a reduction in its volume at term may increase the risk of caesarean section[38]. It is therefore necessary to definitively clarify the efficacy, the most effective strategy with which to administer MH and its clinical significance in improving the AFV in both pregnancies complicated by IO and pregnancies with normal amniotic volume.

The aim of this systematic-literature-review and meta-analysis was to collect all data regarding proposed strategies and their efficacy in relation to each clinical condition for which MH-therapy was performed with the aim of increasing amniotic-fluid (AF) and improving perinatal outcomes. In detail, the primary outcome was to determine, in cases of IO, whether MH-therapy could significantly improve AVF as compared with no treatment, the most effective hydration strategy and most importantly whether such intervention is associated with improvements in perinatal outcomes. Secondary outcome was to evaluate if the AVF variation in IO pregnancies can be comparable to that of patients with normal AFV.

## Materials and Methods

### Data Sources

A systematic literature search (English literature only) was conducted in electronic databases MEDLINE, EMBASE, ScienceDirect and the Cochrane library in the time interval between January 1991 and December 2014.

All studies which evaluated the effects of MH (including oral, intravenous or a combination of both strategies) were collected and analyzed.

### Search strategy

The Key search terms included: Ultrasound/Ultrasonography/Transabdominal sonography [Mesh] and amniotic fluid volume, adding the subsequent subheadings: isolated oligohydramnios OR abnormality in amniotic fluid OR oral fluid intake OR maternal hydration OR intravenous fluid administration OR hypotonic fluid OR water OR improvement in amniotic fluid index OR normal amniotic fluid index OR idiopathic abnormalities in amniotic fluid OR gestational age at diagnosis OR gestational age at delivery OR type of delivery OR neonatal adverse events.

After screening of titles, abstracts and full texts, the selection of included studies was based on the availability of information regarding AFV, type of maternal fluid intake (hypotonic or isotonic), route of fluid administration (oral, intravenous or both), length of treatment (hours, days, weeks), gestational age at diagnosis, gestational age at delivery, type of delivery, and neonatal adverse events.

A second search was performed by using the same criteria but instead searched for AFV changes in patients with normal AFI.

Studies were selected in a 2-stage process. Titles and abstracts from electronic searches were scrutinized by 2 reviewers independently (A.V; ADA) and full manuscripts and their citations list were analyzed by a third reviewer (M.N) to retrieve missing articles and to select eligible manuscripts according to inclusion and exclusion criteria.

### Inclusion/exclusion criteria

We considered eligible all clinical trials comparing the effect of MH between interventional and control groups (no hydration, different route or type of fluid administration, different time of intervention) using a random allocation strategy with adequate allocation concealment and without violations of allocated management or exclusions after allocation which were insufficient to materially affect outcomes. We considered trials which included in the treatment group pregnant women diagnosed with IO in the second half of pregnancy and as controls women with a diagnosis of IO or with normal AFV either receiving no hydration or a different treatment protocol from that of the intervention group (considering volume and type of fluid, route of administration and length of treatment). To be considered eligible, data must be reported clearly and allow for statistical comparison regarding variation in AFV after intervention/no intervention.

We excluded studies in which the diagnosis of oligohydramnios was made by methods different from AFI, when outcomes measured did not minimize observer bias, when missed data influenced conclusions, when no data was available for analysis according to original allocation, or data format was not suitable for analysis.

### Data extraction and management

We designed a form for data extraction. In the eligible studies, two Authors (S.G; T.S.P.) extracted the data using the agreed upon form. We resolve discrepancies through discussion or, if required, by consulting the Authors of the original reports. Data was entered into the pre-installed Data Sheet form of free Excel software extension for meta-analysis (software MetaEasy 2013 version 1.0.5) and checked for accuracy.

Regarding the strategies of maternal hydration we clustered trials and cohorts of patients according to the following outcome measures:

Route of administration: oral versus intravenous versus combination;
Total daily dose of fluid administered: less than 2000 versus more than 2000
Length of hydration treatment: 1 day, more than 1 day but less than 1 week, more than 1 week
Type of fluid administered: isotonic versus hypotonic versus combination of both

### Risk of bias (quality of the included studies)

The methodological quality of each study was evaluated with QUADAS-2 (Quality Assessment of Diagnostic Accuracy Studies 2).[39]

The risk of bias in patient selection, index test, reference standard, flow and timing as well as the concerns for applicability related to the first three domains are shown in S1 Fig. Prisma checklist 2009 version is shown in S1 PRISMA Checklist.

### Endpoints

Primary endpoint was to compare different strategies of MH versus no treatment in terms of Δ variation in amniotic fluid index (AFI), considering only the cohort of patients with diagnosis of IO.

Secondary endpoint was to compare protocols in terms of type of fluid (isotonic versus hypotonic), route of, (intravenous versus oral) and length of fluid administration (single day versus multiple days) in order to evaluate Δ variation in AFI in the cohorts of patients diagnosed with IO.

Tertiary endpoint was to compare different strategies of MH in terms of Δ variation in AFI considering the cohorts of patients with diagnosis of IO versus patients with normo-hydramnios.

Finally we evaluated Δ variation in AFI after different strategies of MH in a cohort of women with normo-hydramnios.

Additionally, when possible, we correlated Δ variation in AFI with mean gestational age at diagnosis, mean gestational age at delivery, type of delivery and percentage of adverse neonatal outcomes considering only the cohort of patients with diagnosis of IO.

### Statistical analysis

The Meta-analysis was conducted according to the Preferred Reporting Items for Systematic Reviews and Meta-Analyses (PRISMA) guidelines using Comprehensive Meta-Analysis software V.2.2 (Biostat, Englewood, New Jersey, USA).

For dichotomous data, we presented results as summary risk ratio with 95% confidence intervals. For continuous data, we used the mean difference if outcomes were measured similarly between trials. We used the standardized mean difference to combine trials that measured the same outcome, but by different methods.

The impact of including studies with high levels of missing data in the overall assessment of treatment was explored by sensitivity analysis. For all outcomes, analysis was carried out, when possible, on an intention-to-treat basis, i.e. we attempted to include all participants randomized to each group in the analysis. The denominator for each outcome in each trial was the number of patients randomized minus any participants whose outcomes were known to be missing. We used the I^2^ statistic to measure heterogeneity among the trials in each analysis. If we identified substantial heterogeneity (I^2^ >50%) we would explore it by pre-specified subgroup analysis.

Where we suspected a reporting bias we attempted to contact study authors requesting missing outcome data. Where not possible, and the missing data was thought to introduce serious bias, the impact of including such studies in the overall assessment of results was explored by a sensitivity analysis.

We used fixed-effect inverse variance meta-analysis for combining data where trials were examining the same intervention, and the trials’ populations and methods are judged sufficiently similar. Where we suspected clinical or methodological heterogeneity between studies sufficient to suggest that treatment effects may differ between trials we used random-effects meta-analysis.

If substantial heterogeneity was identified in a fixed-effect meta-analysis this was noted and the analysis repeated using a random-effects method. We carried out sensitivity analysis to assess the effect of including trials with greater risk of bias, if there were sufficient trials.

## Results

Using the above mentioned key search strategy, we identified 82 potentially relevant papers. Only 16 of the above manuscripts, reporting data collected in a cohort of 1121 pregnant women, were included in this meta-analysis after application of the inclusion and exclusion criteria.[14–29]

Of these 16 articles, 8 met the inclusion criteria for the first endpoint [15,16,18,20,21,25–27], 4 for the second endpoint [14,18,20,22], 2 for the third endpoint [19,24] and 4 for the fourth endpoint [17,23,28,29].

A full report regarding authors, study design, sample size, epidemiological features of studied population, type of intervention and outcome measures is shown in detail in Table 1 and in the flow diagram (S2 Fig).

**Table 1:** Descriptive analysis of trials included in the meta-analysis.

AUTHORS and YEAR	STUDY SETTING [sample size]	PATIENTS & METHODS	RESULTS	GW AT DIAGNOSIS	GW AT DELIVERY	CS RATE	NEONATAL DISTRESS/ADVERSE EVENTS RATE	CONCLUSIONS
***Kilpatrick et al 1991***	RCT [**36**]	*IO (AFI<6 cm)*. **Study Group**: (n = 19) oral hydration 2000 ml/2-4 h. **Control Group** (n = 17): routine hydration (n = 10), routine hydration plus 100 ml water (n = 7)	The mean AFI increased significantly in Study Group (p<0.01).	AT TERM 37±4.8 VS 39±2.4	NR	NR	NR	*Oral MH increases AFI in women with decreased AFV*.
***Kilpatrick et al 1993***	RCT [**40**]	*Normal AFI index (7–24 cm)*. **Study Group**: (n = 20) oral hydration of 2000 ml/2 h **Control Group**: (n = 20) oral hydration of 100 ml/2 h	The mean AFI increased significantly in Study Group (p<0.0001), while decreased in Control Group (p<0.02)	THIRD TRIMESTER [MORE THAN 28]	NR	NR	NR	*MH status plays a role in AVF regulation in women with either normal or decreased amniotic fluid*.
***Doi et al 1998***	RCT [**84**]	*IO (AFI<5 cm)*. **Group A**: (n = 21) intravenous hydration of isotonic fluid 2000 ml/2 h (Lactated Ringer solution) **Group B**: (n = 21) intravenous hydration of hypotonic fluid 2000 ml/2 h (diluted Ringer solution) **Group C**: (n = 21) oral hydration of 2000 ml/2 h. **Control Group**: (n = 21) no hydration	The mean AFI increased significantly Group B and C (p<0.001), but not in Group A.	AT TERM 39.5 VS 39.7 VS 37.3 VS 38.9	NR	NR	NR	*In women with IO*, *a significant increase in the AFI was achieved by both IV hypotonic fluid loading and oral hydration*, *but not by IV isotonic fluid loading*.
***Deka et al 2000***	OBS-PCS [**50**]	*IO (AFI<8 cm)*. **Study Group**: (n = 25) 2000 ml oral hydration in 1 hour **Control Group**: (n = 25) no hydration	The mean AFI increased significantly in all patients after 3 hours (p<0.001)	EARLY PRETERM 28	NR	NR	NR	*Simple oral MH may help to sustain a ‘steady state’ amniotic fluid volume*, *and in the prevention and management of IO during pregnancy and labor*.
***Chandra et al 2000***	RTS [**41**]	*IO (AFI<6 cm)*. **Group A** (n = 16) oral hydration with 10–12 glasses of water per day (61.9±11.7 hours between pre and post-treatment AFI measurements). **Group B** (n = 25) intravenous hydration (Lactated Ringer solution, 45.1±8.9 hours between pre and post-treatment AFI measurements). **Subgroup B1** (n = 15) ≤2000 ml hydration. **Subgroup B2** (n = 10) ≥2500 ml hydration	The mean AFI increased minimally after hydration (51.2% of patients) with no differences between the two Groups. -AFI increase was not related to entity of hydration	AT TERM 38.5±0.39 VS 38.8±0.5	NR	NR	NR	*IO may respond to oral and intravenous hydration*.
***Fait et al 2003***	OBS-PCS [**60**]	*IO (AFI<6 cm)*. **Study Group**: (n = 30) at least 2000 ml oral daily hydration **Control Group**: women with physiological pregnancy (routine hydration).	The mean AFI increased significantly in the Study Group after 1 week (p<0.01).	EARLY PRETERM 29 [RANGE: 26–34] VS 28 [RANGE:26–35]	NR	NR	NR	*Long term oral hydration increase AFI for at least a week*.
***Lorzadeh et al 2008***	RCT [**80**]	*IO*. *(AFI<5 cm)*. **Group A**: (n = 20) intravenous hydration of isotonic fluid 2000 ml/2 h **Group B**: (n = 20) intravenous hydration of hypotonic fluid 2000 ml/2 h **Group C**: (n = 20) oral hydration 2000 ml/2 h (water) **Control Group**: (n = 20) no hydration	The mean AFI increased in Group B and C (p<0.0001) without significant change in Group A.B Delta AFI was greater in Group C, in comparison with A and B Groups (p<0.0001)	AT TERM 39.0 ± 1.3 VS 38.9±1.27	NR	NR	NR	*MH with oral water*, *hypotonic fluid and isotonic fluid increases AFI in I*.*O*. *MH with oral water was more effective than other groups*.
***YanRosemberg et al 2008***	RCT [**44**]	*IO (AFI<6 cm)*. **Group A** (n = 21): 2000 ml iv (1/2 normal saline solution) in 2 h **Group B** (n = 23): placebo (20 ml iv 1/2 normal saline solution in 2 h)	The mean AFI increased significantly in both groups (p<0.05), but not if a comparison was made.	A TERM 39.2±1.2 VS 39.1±1.3	A TERM 40.1+1 VS 40+1.1	10% VS 9%	NR	*Acute intravenous hydration with hypotonic solution did not increase AFI in IO*
***Umber et al 2010***	RCT [**50**]	*IO (AFI<5 cm)*. **Group A**: (n = 25) intravenous hydration of 2000 ml/2 h (5% W/D solution) **Group B**: (n = 25) oral hydration of 2000 ml/2 h	The mean AFI increased significantlyin both Group A and Group B (p<0.05), with no significant differences between the two groups.	THIRD TRIMESTER [RANGE: 28–42]	NR	NR	NR	*Intravenous as well as oral MH increases AFI*, *but neither appears to be particularly advantageous over the other*.
***Borges et al 2011***	RCT [**99**]	*Normal AFI*. **Group A**: (n = 34) oral hydration of 1500 ml/2-4 h of isotonic solution **Group B**: (n = 30) oral hydration of 1500 ml/2-4 h of water **Control Group**: (n = 35) oral hydration of 200 ml/2-4 h of water	The mean AFI increased in Group A and B (p<0.001) and was reduced in controls, but with no significant difference among Groups.	LATE PRETERM 35±1.53 VS 35.4±1.6 VS 34.4±2.2	NR	NR	NR	*In women with normal AFI*, *a significant increase in AFI was achieved by oral hydration with both isotonic solution and water*.
***Patrelli et al 2012***	RCT [**137**]	*IO (AFI<5 cm)*. **Group A**: 66 patients with IO **Subgroup A1** (n = 33): 1500 ml iv (Ringer solution) + 1500 ml oral daily for 6 days. **Subgroup A**2 (n = 33): 1500 ml iv (Ringer solution) + 2500 ml oral daily for 6 days. **Group B**: 71 women with physiological pregnancy (routine hydration).	The mean AFI increased in group A after therapy (P<0.001) without differences between subgroups. The mean AFI at birth was greater in subgroup A2 in comparison to A1 (P<0.001).	EARLY PRETERM 31.5±1.2 VS 31.4±1.3	A TERM 39.5±1.1 VS 39.4± 1.3	30% VS 18%	0% VS 0%	*In pregnancies complicated by IO and treated with intravenous hydration therapy for 6 days the quantity of AF is significantly improved compared to pregnancies not complicated at the same gestational age*.
***Ghafarnejad et al 2012***	RCT [**37**]	*IO (AFI<6 cm)*. **Group A** (n = 22): 2000 ml oral in 2 h + routine hydration for 24 h. **Group B** (n = 22): routine hydration for 24 h	The mean AFI increased in group A after therapy (P<0.001).	LATE PRETERM 35.1±1.4 VS 36±2	NR	10% VS 9%	NR	*Acute oral hydration is a noninvasive*, *easily accessible and cheap way of increasing AFI*, *which should be encouraged*.
***Shahnazi et al 2012***	RCT [**20**]	*IO (AFI<5 cm)* **Study Group**: (n = 10) intravenous hydration of 1000 ml/30 min (isotonic saline solution) **Control Group**: (n = 10) no hydration	The mean AFI increased significantly in Study Group in comparison to controls (p = 0.03).	AT TERM 38.6±1.28 VS 39.37±0.78	NR	30% VS 45.5%	10% VS 27.3%	*MH is recommended as a low-cost method with no complications for the fetus and the mother*.
Akter et al 2012	RCT [**64**]	*IO*. *(AFI<5 cm)* **Study Group**: (n = no data) oral hydration (water) 2 l + routine hydration/day for 7 days **Control Group**: (= no data) routine hydration	The mean AFI increased in Study Group in comparison to controls. (p<0.05)	PRETERM [RANGE: 32–35]	NR	28% VS 78.2%	16.2% VS 71.8%	*Oral MH therapy significantly increases the AFI*, *reduces the caesarean section rate and improves the fetal outcome*.
Ülker et al 2013	RCT [**79**]	*Normal AFI*. **Study Group**: (n = 40) oral hydration of 500 ml before first AFI assessment and 1250 ml before last AFI measurement (left lateral decubitus position) **Control Group**: (n = 39) no hydration (left lateral decubitus position)	The mean AFI increased in both Groups (p<0.05), but did not show any significant difference between the two Groups.	LATE PRETERM 36.32 ± 0.86 VS 36.77 ± 1.46	NR	NR	NR	*Maternal rest in the left lateral decubitus position with hydration and maternal rest in the left lateral decubitus position alone cause similar increases in the estimated AFV*.
Burgos et al 2014	OBS-PCS [**200**]	*Normal AFI*. **Study Group: (n = 100) intravenous hydration** of 2000 ml/2h (hypotonic saline) before the version attempt **Control Group**: (n = 100) no hydration before the version attempt	The mean AFI increased in the Study Group in comparison to controls (p<0.01).	AT TERM [RANGE: 37–41]	NR	37% VS 37%	0% VS 0.1%	*The intravenous option is a safe and effective way to increase the amount of amniotic fluid before external cephalic version*, *with no associated risks for the pregnancy*.

**AFI**: amniotic fluid index, **AVF**: amniotic fluid volume, **IO:** isolated oligohydramnios, **MH**: maternal hydration, **GW**: gestational week, **CS**: cesarean section **RCT**: randomized controlled trial; **OBS:** observational study; **PCS**: perspective controlled study, **NR**: not reported

As above mentioned, the reference standard for single papers and quality of the included studies is illustrated in supplementary S1 Fig.

### Δ variation in AFI index: different strategies of maternal hydration versus no treatment (IO only)

Six studies reported data regarding oral hypotonic fluid administration (276 women), [15,16,18,20,25,27], three studies [18,20,21] reported data regarding intravenous isotonic fluid administration ≥2000ml (126 women) and one study regarding fluid intake <2000ml (20 women)[26], two studies regarding intravenous hypotonic fluid administration (82 women)[18,20]. The analysis of data regarding Δ variation in AFI index in a cohort of women with diagnosis of IO showed significant differences between the intervention group compared to the no treatment group considering both fixed and random effects.[p<0.0001]

**Fig 1:**
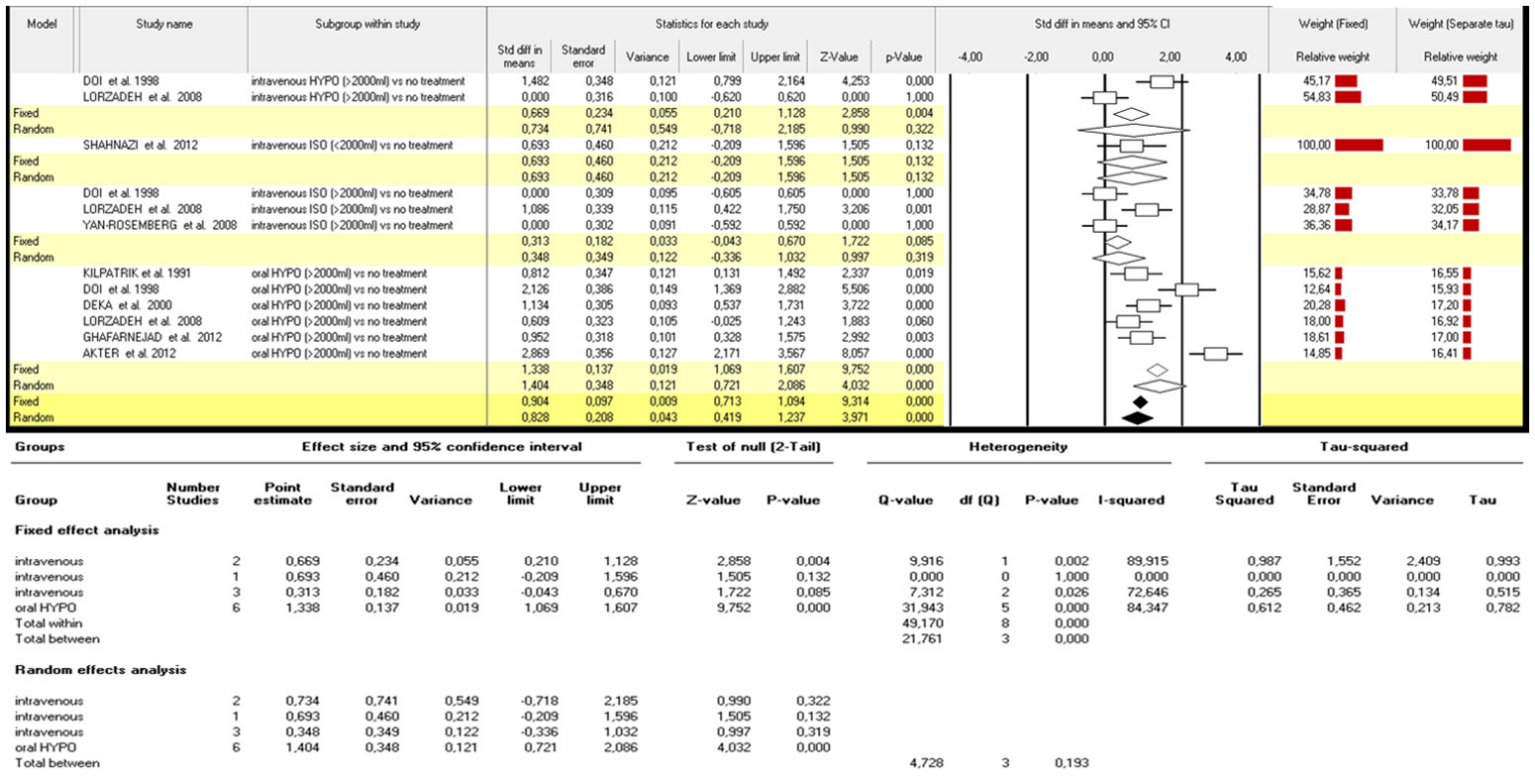
Δ variation in AFI index: different strategies of maternal hydration versus no treatment [isolated oligohydramnios only].

In detail, better results were collected when the intervention group was treated by oral hypotonic fluids (≥2000ml) [p<0.0001] while inferior AFI improvements were noted after intravenous isotonic fluid administration (≥2000ml)[p:n.s]. Contrasting results (despite collected only from 2 studies) were reported after intravenous hypotonic fluid administration (≥2000ml). [p<0.01 in fixed model; p:n.s. in random model] (Fig 1).

### Δ variation in AFI index: different types of fluid intake (isotonic versus hypotonic), hydration strategies (intravenous versus oral) and length of fluid administration (single day versus multiple days) (IO only)

Two studies compared effects of intravenous versus oral hypotonic fluid administration (2000ml) in one day (82 women);[18,20] two studies compared effects of isotonic intravenous versus hypotonic oral fluid administration (2000ml) in one day (90 women); [20,22] one study compared isotonic versus hypotonic intravenous fluid administration (2000ml) in one day (42 women); [18] and one study compared variation in AFI index after 5 days of isotonic intravenous (<2000ml versus >2500ml) plus 1500ml hypotonic oral fluid administration (57 women)[14].

**Fig 2:**
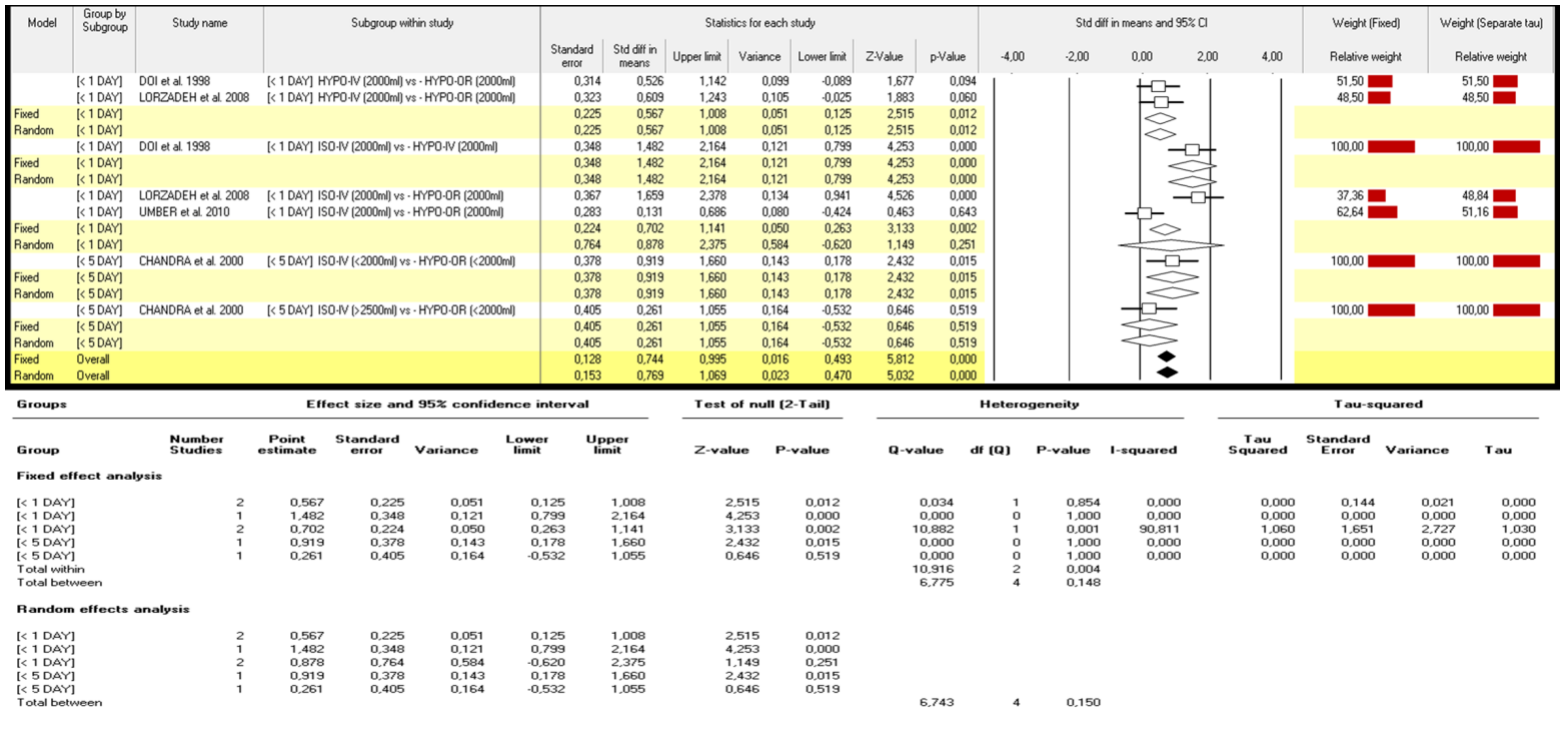
Δ variation in AFI index: different types of fluid intake (isotonic versus hypotonic), hydration strategies (intravenous versus oral) and length of fluid administration (single day versus multiple days) [isolated oligohydramnios only].

The analysis of data showed no significant improvements after the increase in daily dose of isotonic intravenous hydration and no significant differences between treatments completed within one day compared to those which lasted longer than 24 hours.[p:n.s.] The best results were achieved by intravenous hypotonic hydration of 2000ml administered within a single day, despite the fact that data was collected only from a single study [18]. Discordant results were reported regarding the administration of 2000ml of isotonic intravenous fluid versus 2000ml of hypotonic oral administration with significant improvements reported by Lorzadeh et al. [p<0.0001] [20] while no significant improvements were reported by Umber et al[22]. (Fig 2).

### Δ variation in AFI index: different strategies of maternal hydration versus no treatment (IO versus normo-hydramnios)

Two studies compared the effects of different strategies of maternal hydration for amniotic fluid improvement in IO versus untreated matched pregnancies with normo-hydramnios (95 cases versus 101 controls)[19,24].

**Fig 3:**
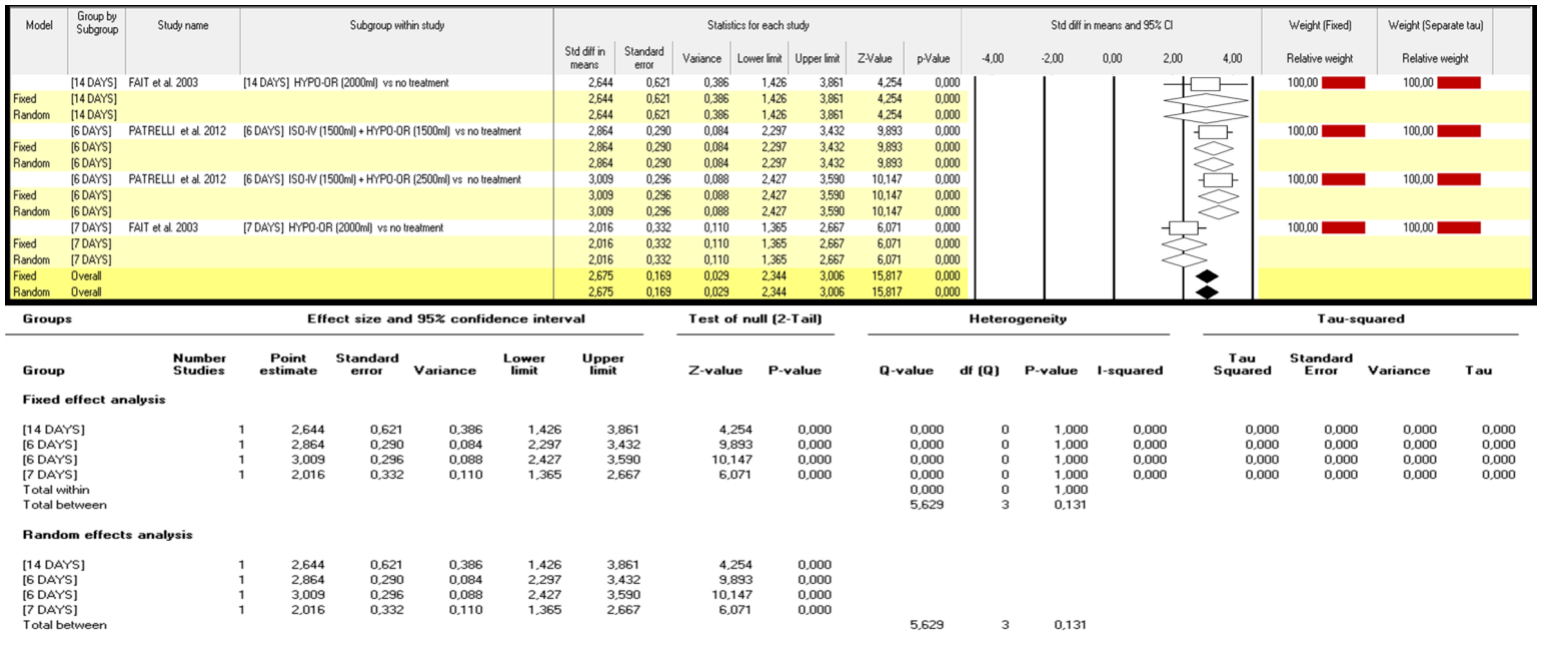
Δ variation in AFI index: different strategies of maternal hydration versus no treatment [isolated oligohydramnios versusnormo-hydramnios].

Patrelli et al. compared a 6 day treatment protocol consisting of isotonic intravenous fluid (1500ml) plus a hypotonic oral fluid intake (1500ml versus 2500ml) to a cohort of untreated controls. Both treatments resulted significantly effective in improving the AFI index [p<0.0001] with no significant differences observed between the two hydration schemes. [24] Interestingly, similar effects were collected by Fait et al. in a cohort of cases treated by a 2000ml intake hypotonic fluid administered orally for 14 days. [19]. The same hydration protocol appeared to be less effective when administered for a period of 7 days (Fig 3).

### Δ variation in AFI index: effects of different strategies of maternal hydration (normo-hydramnios only)

**Fig 4:**
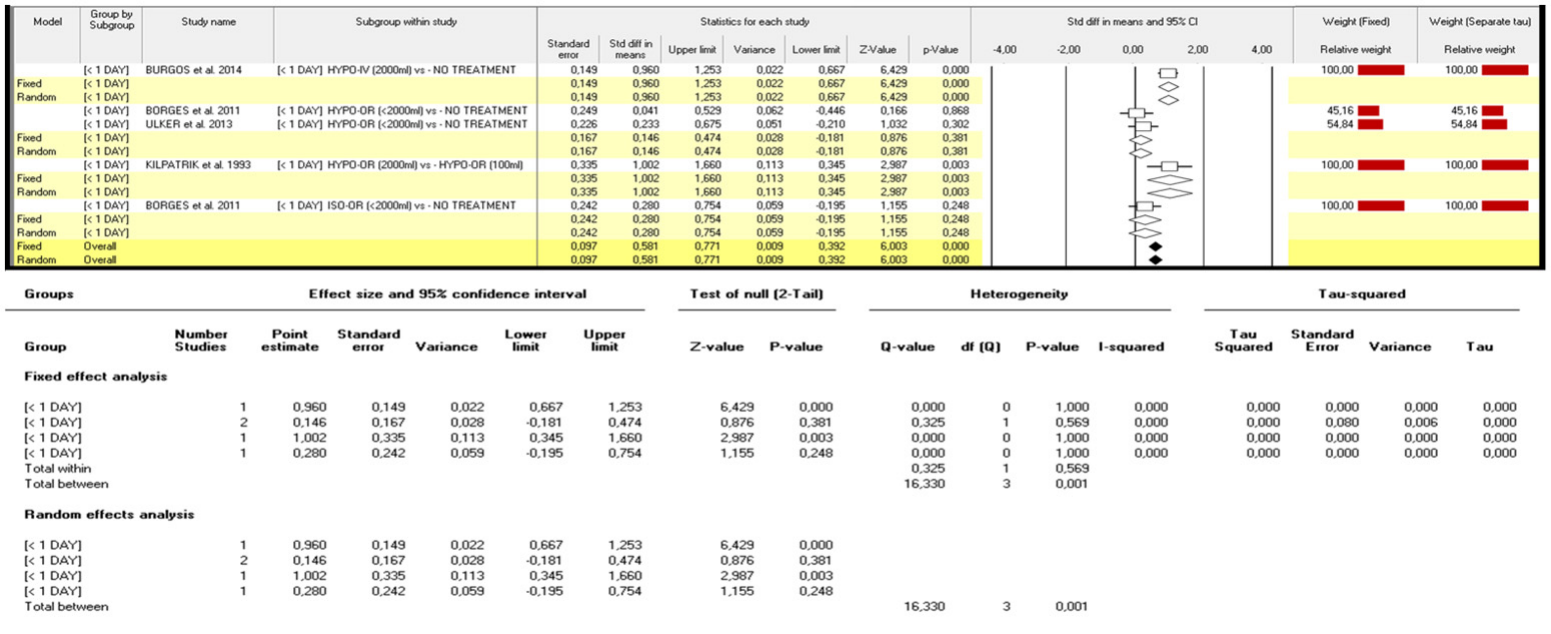
Δ variation in AFI index: effects of different strategies of maternal hydration [normo-hydramnios only].

Four studies compared effects of different strategies of MH for amniotic fluid improvement in pregnant women with normo-hydramnios (453 women), all focused on short-term therapy (within one day).[17,23,28,29]. In detail two studies compared oral hypotonic fluid administration (<2000ml) versus no treatments reporting no differences in term of AFI index variation [p:n.s.].[28,29]A small, though not significant improvement was reported by Borges et al who administered 2000ml of isotonic solution orally [23] while a significant improvement was collected by Burgos et al and Kilpatrik et al [17,29]who administered 2000ml of hypotonic solution intravenously and orally, respectively[p< 0.0001 and p<0.01, respectively] (Fig 4).

Regarding gestational age at diagnosis, as shown in table 1, the majority of manuscripts reported heterogeneous or incomplete data (absence of absolute value, standard deviation or confidence interval). Additionally, only two studies, comparing different outcomes, reported data regarding gestational age at delivery [21,24]. All remaining studies lacked data regarding the time interval between gestational age at intervention and delivery, therefore making statistical correlation studies impossible. Likewise, only 6 manuscripts reported data regarding the mode of delivery and the cesarean section rate [21,24–27,29] but the heterogeneity between the studies made a statistical evaluation of this outcome impossible. The same conditions applied for the evaluation of neonatal outcomes.

## Discussion

Though the ultimate goal of our meta-analysis was to evaluate the actual e effects of MH in both pregnancies with IO and with normal AFV, the majority of eligible studies (10 of 16 trials) merely reported information regarding the variation in AFV following intervention.[14–20,22,23,28] Unfortunately, studies reporting data regarding perinatal outcomes were affected by relevant heterogeneity in outcome measures, data collection, eligibility criteria, and hydration strategy which made it impossible to make comparisons without incurring in bias in statistical evaluation.

The descriptive data reported in Table 1 clearly demonstrated that in all cases of MH neither complications nor adverse events were reported. Interestingly, when hydration was associated with improvements in AFI, it also associated with a significant reduction in cesarean section rates and with a percentage of term deliveries comparable to that of normal pregnancies.

Though the last Cochrane [32], published in 2002, concluded that “since the studies reviewed have not assessed clinically relevant outcomes or possible complications, there is no evidence to support the use of MH in routine practice, except in the framework of further clinical trials designed to address these issues”, the encouraging data from subsequent trials (6 studies evaluating the impact on type of delivery and 4 on neonatal outcomes (see Table 1) allow us to postulate that the possibility of increasing AFV with a simple, non-expensive and less-invasive/non-invasive method such as MH should be considered a useful tool in a modern conception of obstetrical care.

We are conscious that stronger evidences are mandatory in order to introduce guidelines regarding MH intervention even during the third trimester of pregnancy (particularly when an isolated finding) and at term with the scope of improving the outcome of delivery and peri-partum care (favoring spontaneous cephalic version or increasing the success of external rotation maneuvers).

Despite the limitations of the available data (impossibility to evaluate pregnancy, delivery and neonatal outcome after interventions, large confidence interval for gestational age at diagnosis, different cut-offs for diagnosis of oligohydramnios, different interval time of outcome measure-delta AFI) do not allow us to define the real clinical utility of MH, our results may prove useful in helping clinicians and researchers better design future studies aimed at solving e the dilemma regarding the potential utility of MH.

Even if all above mentioned limitations suggest caution in the interpretation of these results, the statistical analysis of data clearly demonstrated the efficacy of MH in pregnancies affected by IO, since all proposed strategies significantly increased the AFV. On the contrary, when considering the efficacy of hydration in term pregnancy with normal AFI, the strength of the evidence seems to fall and dependence on the therapeutic strategy increased. In this last cohort, it seems that both routes of administration (intravenous versus oral) are equivalent while the administration of hypotonic fluids seems more effective than isotonic fluids in increasing AFV. However these results may be related to the small number of trials conducted and to the heterogeneity of both population and protocols.

When considering IO, evidences suggest that oral hydration is more effective than intravenous hydration in increasing amniotic fluid volume. The indirect explanation of this fact may be identified in the duration of treatment. In all trials in which MH lasted longer than one day, the improvements in AFV appeared less dependent on the total volume of fluid administered. This fact suggests that most likely duration is more important than dose. Perhaps intravenous administration of fluids causes a more transitory increase in maternal volume compared to oral hydration and this may explain the lower efficacy of this strategy. On the other hand, while the dose of fluid intake does not seem to significantly affect the overall AFV increase, the type of fluid administered may significantly influence the final outcome. In general, all clinical trials suggested that in IO, regardless of the route of administration (oral versus intravenous versus combined), hypotonic solutions seems to be more effective than isotonic fluids. This fact is probably due to the physiologic homeostasis existing between the maternal and fetal compartment in maintaining a correct fetal AFV. Indeed Flack et al in 1995 demonstrated, by an elegant case-control study, that maternal hydration affects amniotic fluid precisely via increased transplacental passage, rather than affecting fetal production.[40]

Because isotonic solutions have the same concentration of solutes as plasma, infused isotonic solutions do not flow into cells. Rather, they remain within the extracellular fluid compartment and are distributed between the intravascular and interstitial spaces, thus increasing intravascular volume. On the contrary, infusing a hypotonic solution into the vascular system creates a concentration gradient between the fluid compartments. The infusion of hypotonic crystalloid solutions lowers the serum osmolality within the vascular space, causing a fluid shift from the intravascular space towards the intracellular and interstitial spaces. These solutions hydrate cells, although their use may deplete fluid within the circulatory system.

This physiological mechanism may perhaps explain the importance of duration of treatment rather than total dose administered since the passage of hypotonic fluid into the fetal compartment may be facilitated by time, while an increase in dose may cause an increase in maternal kidney function thus decreasing the dose dependent effect and potentially depleting maternal volume. The lack of data regarding follow-up after intervention in pregnancies affected by IO does not allow us to report regarding the time interval in which the increase in amniotic fluid remains clinically useful. Certainly further trials considering this endpoint may be useful in understanding whether the improvements are time limited and possibly define the average interval time in which further intervention is needed to maintain a safe AFV until delivery. The acquisition of this information may improve perinatal care and “demedicalize” delivery thus reducing the rate of unnecessary/avoidable cesarean section and preterm delivery with their associated complications [41–48]. Interpretation of reported data deserves caution since due to the amount of trials included in data analysis it was not possible to extrapolate information without incurring in some bias. In fact, it was not possible to exclude or quantify potential confounders such as maternal rest during hydration, maternal position during AFI assessment, absolute value of AFI at diagnosis (for example: an increase in AFI by 2 cm may be enough to move a patient from the IO to the low-normal group if the original AFI was above 3, whereas it would not significantly affect the clinical impression for AFI <3 cm).

## Conclusion

Though further studies are mandatory to reach sound conclusions, available data suggests that MH may be a safe, well-tolerated and useful strategy to improve AFV especially in cases of IO. Oral hydration should be preferred since it seems te more effective and potentially more feasible, avoiding invasiveness and necessity of hospitalization and potentially increasing patients’ compliance to treatment. Hypotonic solutions should be preferred to isotonic solutions and administered at low dose (about of 1500 ml per day) for long periods (ideally for 2 weeks). In view of many obstetric situations in which a reduced AFV may pose threats, particularly to the fetus, the possibility of increasing AVF with a simple and inexpensive method like MH may certainly have useful clinical applications in obstetric care.

## Supporting Information

S1 Fig Graphical display of study characteristics according to QUADAS II recommendations to report the risk of bias for patient selection and the concerns for applicability of data collected in manuscripts eligible for the meta-analysis. (TIF)

S2 Fig PRISMA 2009 Flow Diagram. (TIF)

S1 PRISMA Checklist PRISMA 2009 Checklist. (DOC)

## Abbreviations

AF: amniotic fluid
AFI: amniotic fluid index
AFV: amniotic fluid volume
IO: isolated oligohydramnios
MH: maternal hydration
