# Supplementary figures and images for: An Update on Maternal Hydration Strategies for Amniotic Fluid Improvement in Isolated Oligohydramnios and Normohydramnios: Evidence from a Systematic Review of Literature and Meta-Analysis
Source: PLoS One. 2015 Dec 11;10(12):e0144334. doi: 10.1371/journal.pone.0144334 (PMC4684238; doi:10.1371/journal.pone.0144334)

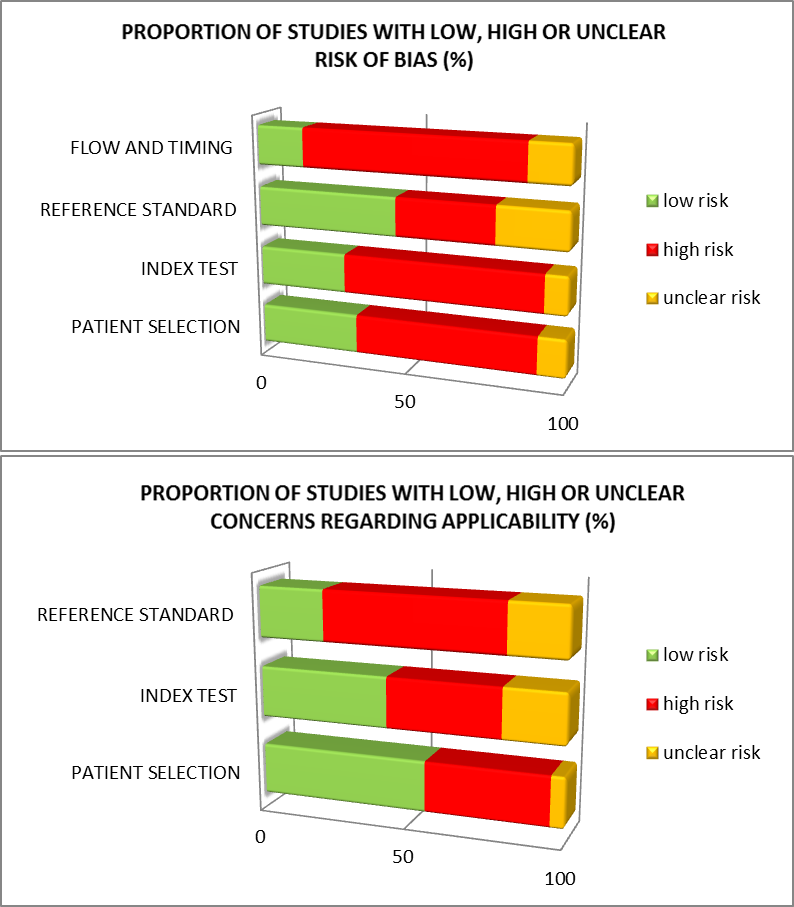

Supplement: S1 Fig — (TIF) [file pone.0144334.s001.tif]

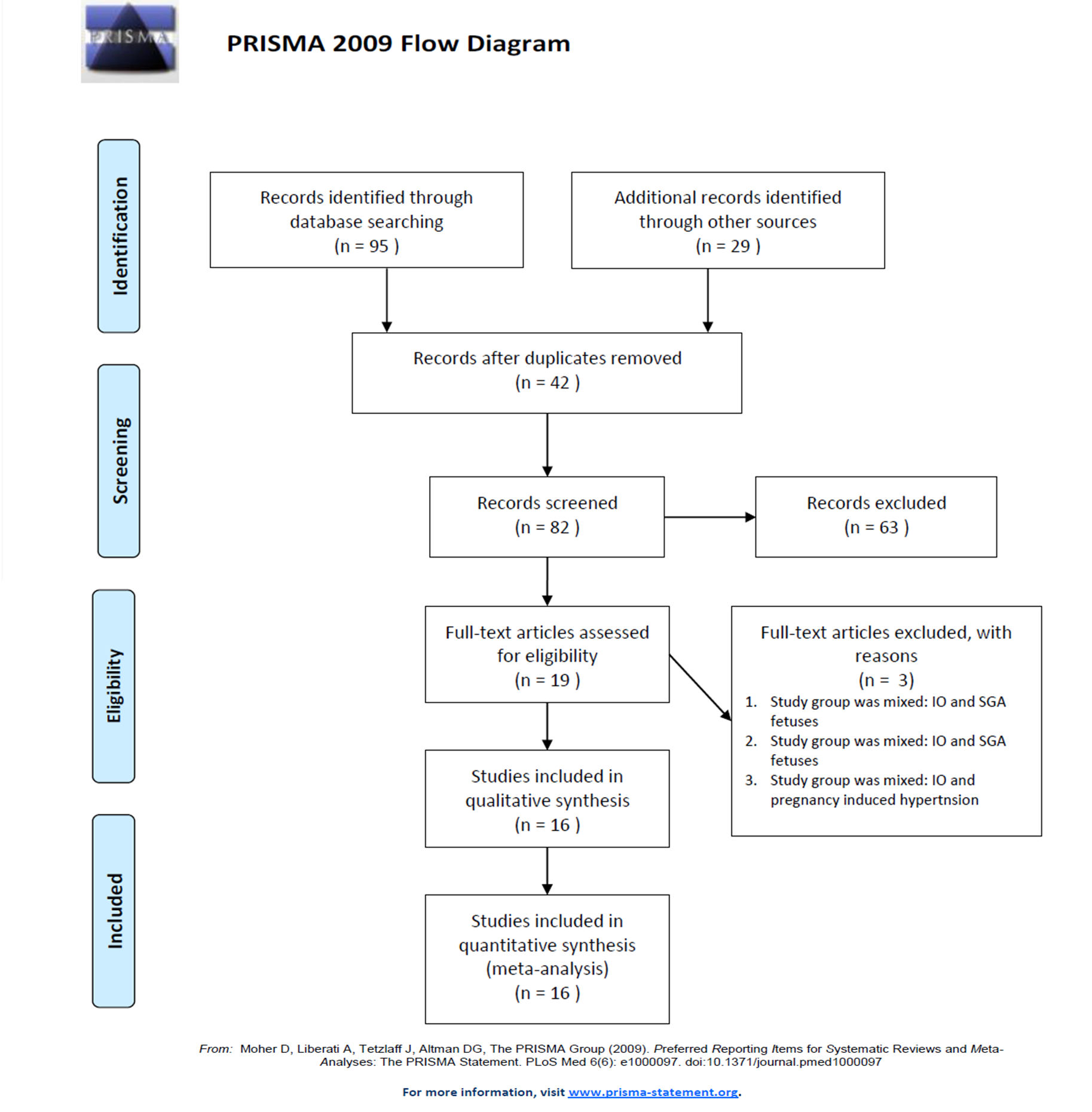

Supplement: S2 Fig — (TIF) [file pone.0144334.s002.tif]
